# Career and Leadership Education in Anesthesia Residency Training

**DOI:** 10.7759/cureus.2546

**Published:** 2018-04-29

**Authors:** David Ninan, Devangi Patel

**Affiliations:** 1 Anesthesiology, Riverside University Health System Medical Center, Moreno Valley, USA

**Keywords:** career, leadership, education, residency, business

## Abstract

Being a well-rounded physician requires competencies that extend beyond traditional medical training. This study explores one residency program’s attempt to address the need to foster career management and leadership skills. A year-long didactic program was initiated to tackle both career management and leadership development. At the conclusion of the program, a survey revealed an increase in perceived competency in the domains taught. There was also a measurable increase in the amount of scholarly and quality improvement projects done in the department where the program was implemented. To support and develop physician competencies, healthcare organizations may derive immense benefit from a program that fosters both career management and leadership skills.

## Introduction

Traditional postgraduate physician education focuses on learning the clinical aspects of a physician’s career. There is a lack of published literature on how training programs address needs such as leadership education and career development [1-5]. A 2017 study of anesthesia residency programs revealed that only 23 of 59 programs had a career development curriculum (CDC) and of those CDCs available, 32.2% were rated as poor or completely ineffective by their program directors [6].

Without a career development curriculum, physicians are left to learn necessary skills through a non-standardized process of ad hoc mentorship, which leads to less-than-optimal career progress for physicians. This lack of career development also leads to a collectively diminished input from physicians in the nation’s health care conversations. There has been a renewed push for incorporating these topics into residency training. In 2017, the Accreditation Council for Graduate Medical Education added requirements specific to these topics [7].

## Materials and methods

Riverside County Regional Medical Center (now named Riverside University Health System) is a 439-bed safety net medical center. It trains resident physicians in several primary and specialty residency training programs. In the2009-2010 academic year, the department of anesthesia implemented an education program to train its residents in the business of medicine and healthcare leadership.

The course was structured around a once-a-month, one-hour didactic program and an assignment to be completed at a medical conference. Each session has an assignment due the day of the session in preparation. The first part of the course focused on the individual’s career management. The latter portion focused on topics important to developing leadership skills.

The career skills covered in the course began with personal branding, curriculum vitae, and resume development. These skills were bundled together and became the building blocks for the rest of the course. An example of an exercise in this course was having residents develop a unique personal brand that would be of interest to a future employer. These courses typically focused on technical expertise in a sought-after area or desirable personality traits, such as being a hard-working team player. Participants were then encouraged to find and complete resume-worthy projects that would highlight their specific areas of strength and provide the additional, collective benefit of increasing the program’s number of quality improvement and academic projects.

The second component was dedicated to marketing residents. More specifically, the course was geared toward helping residents convey their unique values to potential employers. The exercise required that residents write short speeches about their strengths. The students then practiced delivering these speeches to peers who provided feedback to help refine the messaging. 

The next step in their evolution was a networking assignment where they applied what they had learned. Each year, the residency program sends residents to a local medical conference, and each resident is given a networking assignment to complete at the conference. Students are told to engage with a new contact in the form of a casual conversation and practice the script they had refined during the sessions. These skills form a core set of personal skills that are fostered and reinforced throughout the rest of the year’s course.

The next block taught the practical aspects of evaluating and valuating different types of medical practice. It included contracting, insurance, organizational structure, reimbursement, benefit analysis, negotiation and retirement benefits. The final portion of the program covered medical staff structure, governance, health care reform, and future trends in medicine. 

Several measurements were taken to help assess the efficacy of the program. Pre-program and post-program surveys on perceived competency were completed by participants at the first and last sessions, respectively, to get a subjective measure of the program. The survey used a Likert scale. The quantity of scholarly activity generated by the resident physicians was also assessed and compared to the prior two-year average for the program.

In addition to resident-perceived competency, the number of quality improvement and scholarly activity projects was measured before and after implementation of the program. Finally, the number of individuals the resident had contacted because of the networking assignment was measured as an indicator of the application of their networking curriculum.

## Results

The pre-program and post-program survey results on the residents’ perceived competency is presented in Table 1.

**Table 1:** Comparison of resident self-assessment survey results before and after career development and leadership skills program completion CV: curriculum vitae

Objective Measured	Before Program Completion	After Program Completion	Variance
CV Resume Knowledge	3	5	+2
Personal Brand	1	3	+2
Interview Skills	4	5	+1
Networking	1	4	+3
Negotiations	2	4	+2
Practice Valuation	1	4	+3
Benefit Analysis	1	4	+3
Med Staff Structure and Governance	0	5	+5
Healthcare Reform	1	5	+4
Confidence in Job-Finding Skills	1	4	+3
Average Score	1.5	4.3	+2.8

The number of quality improvement projects and scholarly activity per resident increased as well. Before the implementation of this program, there was an average of 0.5 projects per resident. Upon the conclusion of the year, this number increased to an average of 15 projects per resident for that year of training. Finally, the number of residents who had met someone they had not previously known in the field went from none (for the prior three-year average) to 0.75 people/resident.

## Discussion

The implementation of a year-long monthly training program increased the average perceived competencies in the career, business, and leadership domains. While this measure is only in the perceived competency, it likely reflects an improvement in these knowledge domains. One advantage of a written curriculum is that it institutionalizes training and skill-building instead of relying on the disorganized, ad hoc mentorship that previously existed.

In terms of networking, the resulting 75-fold increase in residents who had communicated their value proposition to a stranger in the field cannot be assumed to mean they had achieved a quality network. It did, however, give them an opportunity to practice and reinforce a newly acquired skill with the possible future benefit of building their networks.

From both the trainee’s and the program’s point of view, an additional advantage to a program such as the one described is an increase in the volume of scholarly activity. The quality of the activity was not assessed due to the subjectivity of an analysis like this. This does likely represent an advantage to both the resident and the program as a whole.

One area that was not assessed was the residents’ overall satisfaction with this type of program. Anecdotally, residents in the earlier years of their education expressed subjective frustration that the program was taking them away from their clinical studies. The more senior residents did not express this same frustration. Further, upon follow-up with graduates of the program, they all expressed an appreciation for learning these types of skills. Additional studies and program development efforts based on alumni experience would be of value in the future.

## Conclusions

The implementation of a curriculum focused on developing the resident career, and leadership skills may improve a resident’s perceived knowledge, increase his or her professional network, and increase the volume of scholarly activity by the resident. It is plausible that healthcare organizations may derive immense benefit from a program that fosters both career management and leadership skills, and physicians with this additional training have an improved chance for optimizing their careers, which, collectively, is to the benefit of the healthcare system.
